# The association of multimodal analgesia and high-risk opioid discharge prescriptions in opioid-naive surgical patients

**DOI:** 10.1186/s13741-021-00230-3

**Published:** 2021-12-15

**Authors:** Erica Langnas, Rosa Rodriguez-Monguio, Yanting Luo, Rhiannon Croci, R. Adams Dudley, Catherine L. Chen

**Affiliations:** 1 Department of Anesthesia and Perioperative Care, University of California, San Francisco, 513 Parnassus Ave, S455, San Francisco, CA 94143 USA; 2 Department of Clinical Pharmacy, University of California, San Francisco, San Francisco, USA; 3 Medication Outcomes Center, University of California, San Francisco, San Francisco, USA; 4 Philip R. Lee Institute for Health Policy Studies at the University of California, San Francisco, San Francisco, USA; 5 UCSF Health Informatics, University of California, San Francisco, San Francisco, USA; 6 Department of Medicine, University of Minnesota, Minneapolis, USA

**Keywords:** Opioids, Multimodal analgesia, Oral morphine equivalents, Postoperative pain, Prescribing practices

## Abstract

**Background:**

Opioids and multimodal analgesia are widely administered to manage postoperative pain. However, little is known on how improvements in inpatient pain control are correlated with high-risk (> 90 daily OME) discharge opioid prescriptions for opioid naïve surgical patients.

**Methods:**

We conducted a retrospective observational study of adult opioid-naïve patients undergoing surgery from June 2012 through December 2018 at a large academic medical center. We used multivariate logistic regression to assess whether multimodal analgesic drugs consumed in the 24 h prior to discharge was associated with a reduction in high-risk opioid discharge prescriptions. We identified other risk factors for receiving a high-risk discharge opioid prescription.

**Results:**

Among the 32,511 patients, 83% of patients were discharged with an opioid prescription. In 2013, 34.1% of patients with a discharge opioid prescription received a high-risk prescription and this declined to 17.7% by 2018. Use of multimodal analgesic agents during the final 24 h of hospitalization increased each year, with over 80% receiving at least one multimodal analgesic agent by 2018. The median OME consumed in the 24 h prior to discharge peaked in 2013 at 31 and steadily decreased to 19.8 by 2018. There was a significant association between the use of acetaminophen in the 24 h prior to discharge and a high-risk prescription at discharge (*p* < 0.01). OMEs consumed in the 24 h prior to discharge was a significant predictor of receiving a high-risk discharge prescription, even at low doses. Other factors associated with receipt of a high-risk discharge opioid prescription included male gender, race, history of anxiety disorder, and discharge service.

**Discussion:**

Use of multimodal analgesia regimens in hospitalized surgical patients in the 24 h prior to hospital discharge increased between 2012 and 2018. Simultaneously, opioid use prior to hospital discharge decreased. Despite these gains, approximately one in five discharge prescriptions was high-risk (> 90 daily OME). In addition, we found that prescribing of discharge opioids above inpatient opioid requirements remains common in opioid naive surgical patients.

**Conclusion:**

Providers should account for pre-discharge opioid consumption and use of multimodal analgesia when considering the total and daily OME’s that may be appropriate for an individual surgical patient on the discharge opioid prescription.

**Supplementary Information:**

The online version contains supplementary material available at 10.1186/s13741-021-00230-3.

## Introduction

In 2018, 15% of the US population filled at least one opioid prescription and about one-third of prescription opioids were obtained from surgeons (Centers for Disease Control and Prevention, 2019; Levy et al., 2015). Significant variation in opioid discharge prescribing occurs after surgery, leading to opioid prescriptions filled but ultimately left unused (Hill et al., 2017; Thiels et al., 2018). These wide variations in opioid prescribing suggest that efforts to reduce inpatient opioid consumption do not always translate into reductions in opioids prescribed at discharge (Bates et al., 2011). Excess opioids create a risk for misuse or dependence for patients, and unused opioids may be diverted into the community (Hupp, 2016).

The management of acute pain represents a critical opportunity to reduce opioid use in surgical patients. Multimodal analgesia has been shown to decrease inpatient opioid consumption for many surgical procedures (Hurley et al., 2006; Straube et al., 2005). Current pain management guidelines recommend the use of multimodal analgesic approaches in surgical patients whenever possible (Anesthesiology, 2012). It remains unclear if the opioid-sparing effects of multimodal analgesia in surgical patients are reflected in the opioid prescriptions provided at discharge.

To assess the relationship between multimodal analgesia and discharge opioid prescribing, we performed a retrospective observational cohort study using electronic medical record (EMR) data of patients having inpatient surgery at a large academic medical center between 2012 and 2018. We hypothesized that there was an increase in multimodal analgesia during the study period resulting in reductions in opioid consumption in the inpatient setting and a reduction in opioids prescribed at discharge. In addition, we investigated whether the pre-discharge pain regimen, which includes both opioid and non-opioid approaches, was associated with high-risk (> 90 daily OME) discharge opioid prescriptions after surgery.

## Methods

### Study design and data source

We conducted a retrospective observational cohort study of adult opioid-naïve patients undergoing inpatient surgery from June 2012 through December 2018 at the University of California San Francisco Medical Center. This study was approved by the UCSF IRB, which waived patient consent for acquisition of data (IRB# 18-26728). Data was obtained by retrospective database queries of the UCSF electronic medical record (Epic Systems, Verona WI).

After extraction from an electronic data warehouse, the data were validated for accuracy with iterative chart auditing. To ensure accurate and complete data extraction, data reports were evaluated to identify inconsistencies, missingness, extreme values, and invalid codes. Discrepancy management included reviewing discrepancies, investigating the reason, and resolving them. The data extracted had no missingness. After a proper quality check and assurance, the final dataset was locked so that the dataset could not be modified and only the final clean dataset was used for analysis

### Study cohort

Our study cohort was comprised of opioid-naïve patients aged 18 years and older who underwent surgery requiring a post-operative stay of at least 24 h after discharge from the post-anesthesia care unit (PACU), and who were ultimately discharged to either home, a skilled nursing facility or rehabilitation facility. We defined opioid-naïve as any patient without an active opioid prescription documented in their electronic medical record (EMR) starting 6 months prior to admission.

### Multimodal analgesia

Multimodal analgesia refers to the administration of drugs that act by different mechanisms to reduce opioid requirements and opioid-related adverse effects when treating pain (Anesthesiology, 2012; Rosero & Joshi, 2014). These medications can be administered via the same route or different routes (i.e., oral, intravenous, perineural). For this study, we identified multimodal analgesia as the administration of acetaminophen, NSAIDS, gabapentinoids, or nerve blocks (neuraxial or peripheral) with active infusions in the 24 h prior to discharge.

### Opioid dose calculation

To compare pre- and post-discharge opioid use, we converted all IV, PO, regional, and neuraxial opioid consumed in the 24 h leading up to hospital discharge into oral morphine milligram equivalents (OME) using the 2018 UCSF Pain Management Committee’s opioid equivalent algorithm (University of California San Francisco, Pain Management Committee’s, 2018). The opioid dosage on the discharge opioid prescription was also converted into OMEs using the same opioid conversion ratios. The conversion to daily OMEs included the medication type, dose, route, frequency, and total number of pills in the discharge prescription. The daily dose on the discharge opioid prescription was defined as the maximum allowable dose in a 24-h period according to the written prescription.

### Definition of high-risk prescription

The risk of opioid-related adverse effects is directly associated with the maximum daily oral morphine equivalents prescribed (Bohnert et al., 2011; Brat et al., 2018). Consistent with CDC recommendations, we defined a high-risk prescription as a discharge opioid prescription exceeding 90 OME per day, which has been associated with an increased risk of opioid-related adverse effects, including overdose death (Dowell et al., 2016).

### Covariates

We assessed other variables that may be associated with the discharge opioid prescription, including patient demographic characteristics, history of substance use disorder, depression, anxiety, discharge service, year of surgical admission, hospital length of stay (LOS), and returning to the operating room during the admission. Comorbid conditions were identified using ICD-9 and ICD-10 diagnosis codes listed in the diagnosis fields or the patient problem list (Table S1).

### Statistical analysis

**Fig. 1:**
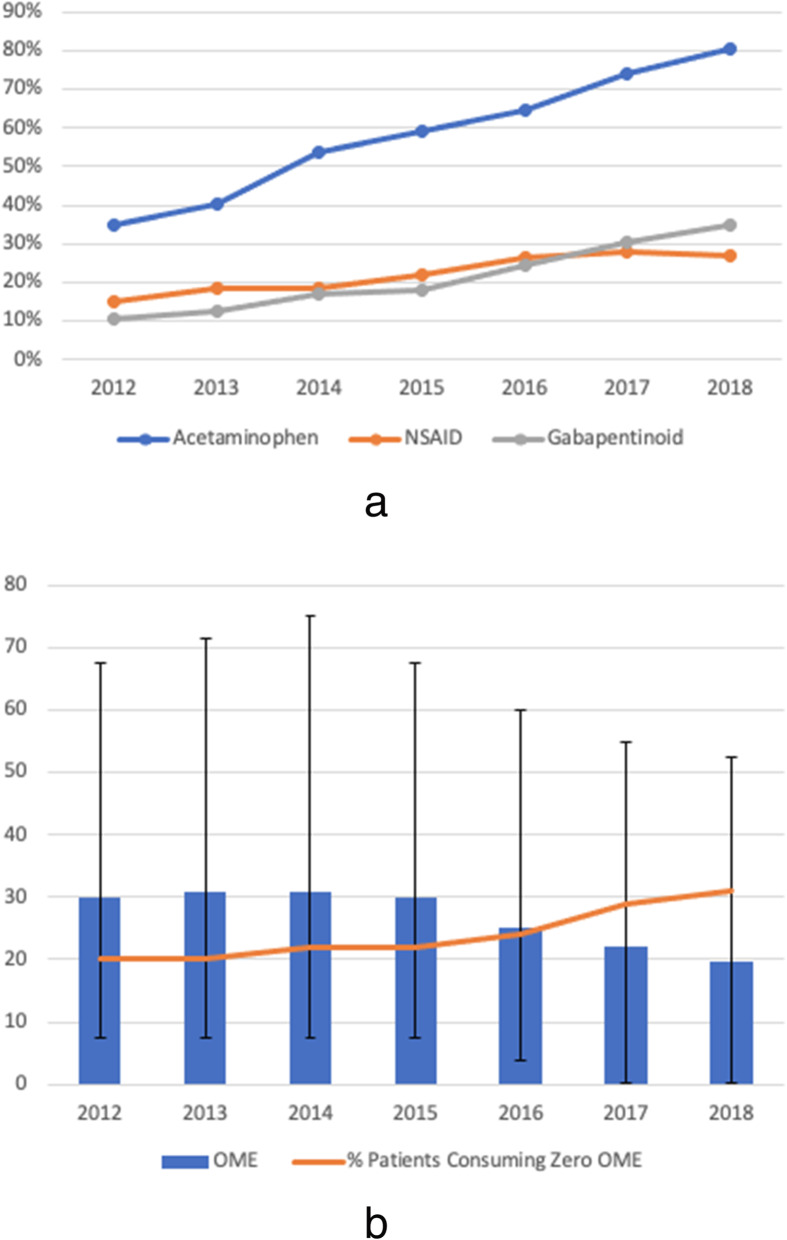
Opioid and non-opioid pain medication consumption 24 h prior to discharge by year. **a** Percentage of patients who consumed acetaminophen, non-steroidal anti-inflammatory drugs (NSAID), or gabapentinoid in the 24 h prior to discharge by year. **b** Median and interquartile ranges of oral morphine equivalents (OME) consumed in 24 h prior to discharge by year and percent of patients consuming zero opioids in the 24 h prior to discharge

**Fig. 2:**
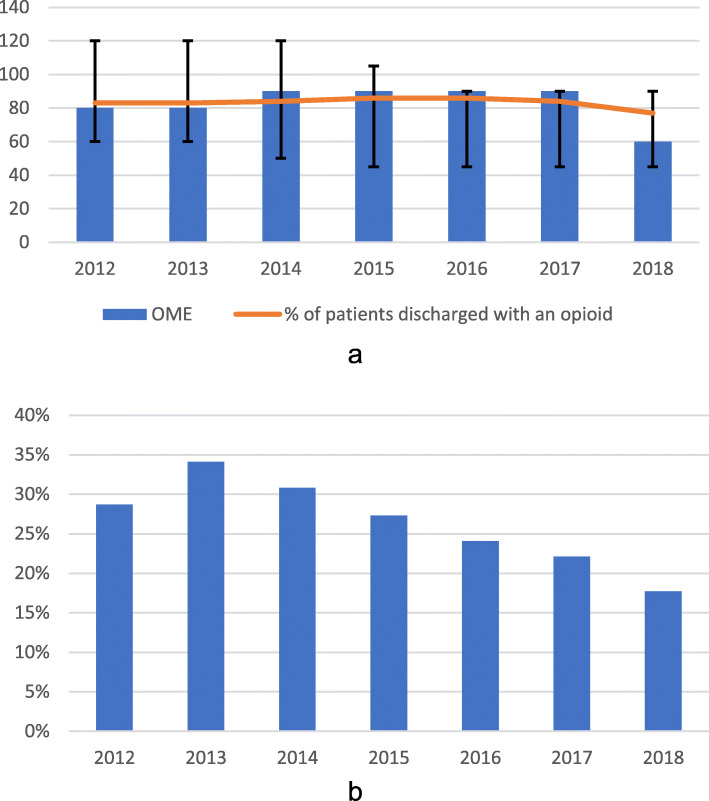
Opioids prescribed at discharge by year. **a** Percent of patients discharged with an opioid and median and interquartile ranges of daily oral morphine equivalents (OME) on discharge prescription by year. **b** Percent of discharge prescriptions that are high-risk (> 90 daily OME)

**Fig. 3:**
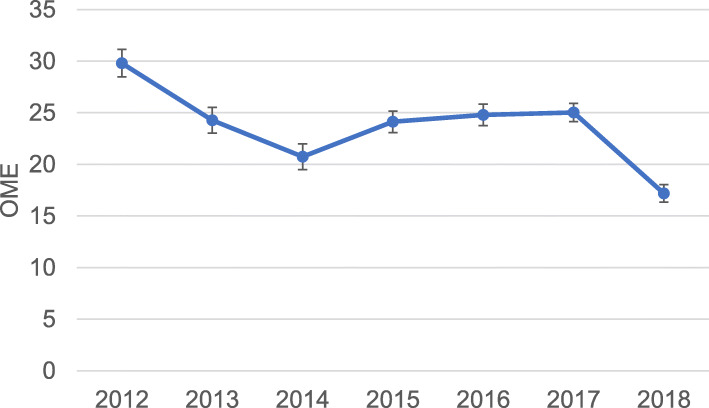
The mean difference, +/− standard error of the mean of daily oral morphine equivalents (OME) on discharge prescription compared to OME consumed in the 24 h prior to discharge by year

We assessed the relationship between each patient’s 24-h pre-discharge pain regimen and the quantity of opioid and non-opioid analgesics prescribed at hospital discharge. We measured inpatient multimodal analgesia use and inpatient opioid consumption in the 24 h prior to discharge from 2012 to 2018 (Fig. 1a and b, respectively). We calculated the daily OME written on the discharge prescription and the percent of patients receiving a discharge opioid prescription between 2012 and 2018 (Fig. 2a). We identified the percent of discharge opioid prescriptions with a dose exceeding 90 daily OME between 2012 and 2018 (Fig. 2b). We compared the dose written on the discharge opioid prescription with the total OME consumed in the 24 h prior to discharge between 2012 and 2018 (Fig. 3). We also identified patients who were prescribed opioids at hospital discharge who did not take any opioid analgesics during their last 24 h of hospital stay. Means, medians, and interquartile ranges (IQR) were used to visualize annual trends in multimodal analgesia, opioid consumption, and opioid prescribing patterns. We used multivariable logistic regression analysis to assess whether multimodal analgesic drugs consumed in the 24 h prior to discharge was associated with a reduction in high-risk (> 90 daily OME) discharge opioid prescribing. We also identified other potential predictors for receiving a high-risk discharge opioid prescription (Table 2).

## Results

We identified 32,511 patients meeting inclusion criteria during the study period. The mean age was 55 years (SD 17), and 47.6% were male. The median LOS was 3.4 days (IQR 2.21, 6.25). Overall, 83% of patients were discharged with an opioid prescription (Table 1).

**Table 1:** Demographic and clinical attributes of study cohort

	Patient encounters (*n* = 32,511)	
Age, mean (sd)	55 (17)	
Gender	%	*n*
male	47.60%	15459
female	52.50%	17050
Race	%	*n*
White	60.20%	19572
Black	5.32%	1730
Asian	13.63%	4431
American Indian or Alaska Native	0.53%	172
Native Hawaii or Pacific Islander	1.27%	413
other	16.48%	5358
unknown	2.57%	835
Surgical admissions per year	%	*n*
2012	6.62%	2153
2013	13.10%	4259
2014	13.20%	4292
2015	15.52%	5045
2016	16.51%	5367
2017	17.37%	5647
2018	17.68%	5748
Length of stay in days, median (IQR)	3.42 (2.21–6.25)	
Return to operating room during surgical admission	7.76%	2522
Epidural or peripheral nerve catheter 24 h prior to discharge	6.77%	2201
Patients discharged with opioid prescription	83.12%	27025

### Pain medication consumption prior to discharge

The median OME administered in the 24 h prior to discharge peaked in 2013 at 31 OME (IQR 7.5, 71.5) and steadily decreased to 19.8 OME by 2018 (IQR 0, 52.5) (Fig. 1b). Use of multimodal analgesic agents during the final 24 h of hospitalization increased every year and peaked in 2018, with 80.3%, 34.1%, and 27.1% of patients consuming acetaminophen, gabapentinoids, or NSAIDs, respectively (Fig. 1a). Excluding patients with nerve catheters or epidurals, 20.2% of patients did not consume any opioid analgesics in the 24 h prior to hospital discharge in 2012. This percentage increased to 30.62% by 2018 (Fig. 1b).

### Pain medication prescribed at discharge

The percent of patients being discharged with an opioid peaked in 2016 at 85.8% of patients, and decreased to 77.3% in 2018, while daily OME on the discharge prescription peaked in 2014 with a median of 90 (IQR 50,120) and decreased to 60 (IQR 45,90) in 2018 (Fig. 2a). In 2013, 34.1% of patients with a discharge opioid prescription received a high-risk prescription; this percentage declined to 17.7% by 2018 (Fig. 2b). The mean difference between total daily OME on discharge prescription and total daily OME consumed in the prior 24 h was positive each year. In other words, more daily OMEs were written on the discharge prescription than consumed in the day prior to discharge in each year of our study. The mean difference peaked in 2012 with a mean of 30 additional OME in the discharge prescription and decreased to 17 by 2018 (Fig. 3). We found that 58.7% of the 8052 patients who did not use opioids or nerve catheters in the last 24 h prior to discharge were prescribed opioids at hospital discharge. The median daily OME written on the discharge prescription for this cohort was 30 (IQR 0.80). Of the patients who did not use opioids or nerve catheters in the last 24 h, 10% received a high-risk discharge prescription of greater than 90 daily OME.

### Predictors for high-risk opioid prescription at discharge

Consumption of gabapentinoids or NSAIDs prior to discharge was not associated with a reduction in the likelihood of receiving a high-risk opioid prescription (Table 2). There was a significant association between the use of acetaminophen in the 24 h prior to discharge and a high-risk prescription at discharge [(AOR), 1.108; 95% CI, 1.041–1.170]. Discharge prescription of acetaminophen was also associated with an increased likelihood of a high-risk discharge opioid prescription [(AOR) 1.104; 95% CI 1.029–1.173], along with discharge prescription of gabapentinoids [(AOR) 1.486; 95% CI, 1.42–1.54]. Total OMEs consumed in the 24 h prior to discharge was a significant predictor of receiving a high-risk discharge prescription, even at low doses. Other patient level factors associated with receipt of a high-risk discharge opioid prescription included male gender [(AOR) 1.132; 95% CI, 1.060–1.209], race, and history of anxiety disorder [(AOR) 2.64; 95% CI, 1.414–4.868]. The discharge services with the highest likelihood of writing high-risk discharge prescriptions were orthopedics [(AOR) 4.662; 95% CI, 4.138–5.252] and thoracic surgery [(AOR) 3.122; 95% CI, 2.450–3.978]. Additional significant predictors included discharge year, with 2013 having the highest association with a high-risk prescription ([AOR] 3.189, 95% CI, 2.818–3.610], and patient length of stay, with length > 6 days associated with a high-risk prescription [(AOR) 2.158; 95% CI, 1.968–2.366].

**Table 2:** Predictors of receiving a high-risk discharge opioid prescription

	AOR	Lower CL	Upper CL	*P* value
Sex (reference: female)				
Male	1.132	1.06	1.209	0.0032
Race (reference: White)				
Black	0.782	0.675	0.907	0.0004
Asian	0.837	0.754	0.928	
American Indian or Alaska Native	0.739	0.486	1.124	
Native Hawaiian or Pacific Islander	0.882	0.651	1.195	
other	0.907	0.831	0.991	
unknown	0.854	0.701	1.041	
Discharge year (reference: 2018)				
2012	2.405	2.072	2.791	< .0001
2013	3.189	2.818	3.61	
2014	2.507	2.218	2.833	
2015	2.355	2.092	2.651	
2016	1.991	1.772	2.237	
2017	1.722	1.534	1.934	
Length of stay in days (reference < 3)				
3 to 6	1.837	1.702	1.983	< .0001
> 6	2.158	1.968	2.366	
History of substance use disorder	0.971	0.734	1.284	0.8348
History of depression	0.942	0.825	1.075	0.3744
History of anxiety	2.625	1.415	4.868	0.0022
Peripheral nerve catheter or epidural used 24 h prior to discharge	1.027	0.916	1.151	0.6502
Multimodal consumed 24 h prior to discharge (reference = no)				
acetaminophen	1.108	1.041	1.170	0.0021
NSAID	1.016	0.927	1.113	0.7392
gabapentinoids	1.027	0.92	1.146	0.6324
Multimodal prescribed at discharge (reference = no)				
acetaminophen	1.104	1.029	1.173	0.0075
NSAID	0.927	0.813	1.056	0.2545
gabapentinoids	1.486	1.420	1.544	< .0001
Total OME consumed 24 h prior to discharge (reference = 0)				
> 0–30	1.577	1.421	1.75	< .0001
30–60	3.057	2.749	3.4	
60–90	5.141	4.58	5.77	
90+	10.431	9.332	11.659	
Days of opioids on discharge prescription (reference ≤ 7 days)				
> 7 days	0.675	0.628	0.726	< .0001
Return to operating room during surgical admission	0.917	0.812	1.036	0.1658
Discharge Service (reference General surgery)				
Orthopedics	4.662	4.138	5.252	< .0001
Thoracic surgery	3.122	2.45	3.978	
Cardiac surgery	2.766	2.304	3.32	
Urology	2.165	1.897	2.472	
Critical care medicine	1.832	0.737	4.557	
Neurosurgery	1.609	1.442	1.794	
Gynecologic oncology	1.368	1.115	1.679	
Malignant hematology	1.278	0.717	2.278	
Advanced heart failure	0.879	0.263	2.94	
Vascular surgery	0.874	0.707	1.081	
Otolaryngology, head and neck surgery	0.837	0.682	1.028	
Advanced lung disease	0.788	0.156	3.988	
Plastic surgery	0.783	0.581	1.055	
Colorectal surgery	0.756	0.606	0.943	
Gynecology	0.695	0.46	1.051	
Hospital medicine	0.549	0.412	0.733	
Transplant surgery	0.516	0.305	0.871	
Liver transplant	0.457	0.354	0.592	
Pediatric service	0.449	0.301	0.671	
Obstetrics	0.404	0.32	0.509	
Kidney transplant	0.397	0.322	0.49	
Neurovascular	0.275	0.096	0.788	
Ophthalmology	0.178	0.023	1.353	
Neurology	0.131	0.032	0.54	
Oral and maxillofacial surgery	0.128	0.052	0.316	
Cardiology	0.059	0.008	0.427	

## Discussion

We found a significant increase in the use of multimodal analgesia regimens in the 24 h prior to discharge in the years 2012–2018, and this trend coincided with a reduction in 24 h pre-discharge opioid consumption, and a reduction in high-risk opioid prescriptions. These findings are consistent with prior studies investigating the effects of multimodal in surgical patients (Hurley et al., 2006; Straube et al., 2005; Militsakh et al., 2018). However, despite these reductions, almost 80% of patients still received an opioid prescription at hospital discharge. In addition, we identified a gap between the gains in inpatient pain management and the gains of safer opioid prescribing practices at hospital discharge in the surgical patient population. Our results showed that approximately one-fifth of patients received a high-risk prescription known for causing opioid-related adverse events.

Prior studies comparing opioid use in the 24 h pre-discharge to discharge opioids have not explored the potential opioid-sparing effect of multimodal analgesia (Chen et al., 2018). Interestingly, our study found that acetaminophen, both consumption prior to discharge and inclusion on the discharge prescription, as well as gabapentinoid prescription at discharge, were associated with an increased likelihood of a high-risk discharge prescription. We suspect that patients who have more pain or require more opioids prior to discharge are being more aggressively optimized by adding adjuncts to help mitigate opioid needs. Other potential explanatory factors that increased the odds of receiving a high-risk prescription included discharge service, male gender, white race, prolonged length of stay, and having a history of anxiety.

The risk for an opioid related adverse event is directly related to the dose prescribed (Bohnert et al., 2011; Brat et al., 2018). Notably, patients who consumed even low doses of opioids prior to discharge were also more likely to receive a high-risk opioid prescription, and this risk increased in a dose-dependent fashion according to the amount consumed immediately prior to discharge. Therefore, initiatives aimed at reducing inpatient opioid consumption combined with tools that promote patient specific opioid prescriptions may have the potential to reduce unnecessary high-risk discharge opioid prescribing as well. Any effort to reduce inpatient opioid consumption should ensure that pain is appropriately managed with a balanced combination of both opioid and non-opioid analgesics.

Our findings highlight lack of consistency in opioid prescribing after surgery in opioid-naïve patients. We found 58.7% of patients who consumed no opioids in their last 24 h prior to discharge and had no nerve block still received opioids. Patients received excess opioids on the discharge prescription, averaging 24 daily OMEs more than what was consumed during their last day of hospitalization. This disconnect between inpatient opioid requirements and the amounts written on the discharge opioid prescription suggests that prescribers have not adapted their discharge prescribing practices to account for individual patient opioid needs after discharge, and these prescribed amounts may be driven by other factors.

A variety of factors affect prescribing behaviors, including diagnostic skills, clinical judgment, drug knowledge, institutional protocols and policies, financial incentives, and motivation to remain up to date on medical practices, which are variable among practitioners (Stern & Trajtenberg, 1998). To address these complex variations in opioid prescribing practices, studies have identified benefits of individualized opioid prescribing and as a result consensus statements have emerged emphasizing patient-centered approaches for discharge opioid prescriptions to reduce over prescribing for surgical patients (Levy et al., 2021). Future initiatives and policies should focus on providing an individualized approach in determining discharge pain medications for surgical patients in order to provide safer opioid prescriptions and avoid inadvertent harm.

### Strengths and limitations

Strengths of this study include the large sample size spanning 6 years of surgical prescribing across all surgical specialties at our academic hospital system and the inclusion of a variety of multimodal analgesic approaches, which have not been addressed in prior studies on discharge opioid prescribing in surgical patients. However, as a single-center study, the findings may not represent prescribing practices at non-academic hospitals or different geographical regions. Finally, the retrospective observational study design using electronic health records limits our ability to assert a causal relationship between the explanatory factors in our model and high-risk discharge opioid prescribing practices. In addition, our study was conducted during a time of increasing evidence of harm associated with unsafe opioid prescribing practices along with the introduction of national, state, and institutional level opioid prescribing initiatives, which may have contributed to the changes in opioid prescribing practices we found. Despite these limitations, this study sheds light on several important discrepancies between opioid-sparing inpatient pain management recommendations and discharge opioid prescribing practices in surgical patients.

## Conclusion

Use of multimodal analgesia regimens in hospitalized surgical patients in the 24 h prior to hospital discharge increased between 2012 and 2018. Simultaneously, opioid use prior to hospital discharge decreased. Despite these gains, approximately one in five discharge prescriptions was high-risk (> 90 daily OME), and prescribing discharge opioids with a total OME above the patient’s inpatient opioid requirement remained a common occurrence. Providers should account for pre-discharge opioid consumption and the concomitant use of multimodal analgesia when considering the total and daily OMEs on the discharge prescription for individual surgical patients.

## Supplementary Information

**Additional file 1: Table S1**: ICD codes for depression anxiety and substance use disorder

## Data Availability

Raw large-scale electronic medical record data were generated at our institution. Derived data supporting the findings of this study are available from the corresponding author on reasonable request with permission of IRB.

## Abbreviations

OME: Oral morphine equivalent
PACU: Post anesthesia care unit
EMR: Electronic medical record
NSAID: Nonsteroidal anti-inflammatory drug
