# Supplementary material for: The association of multimodal analgesia and high-risk opioid discharge prescriptions in opioid-naive surgical patients
Source: Perioper Med (Lond). 2021 Dec 15;10:60. doi: 10.1186/s13741-021-00230-3 (PMC8672612; doi:10.1186/s13741-021-00230-3)
Supplement: Supplementary file 1 — Additional file 1: Table S1: ICD codes for depression anxiety and substance use disorder [file 13741_2021_230_MOESM1_ESM.docx]

Table S1: ICD codes for depression anxiety and substance use disorder.

| **Condition** | **DSM Diagnostic Codes**  **(ICD-10-CM)** | **DSM Diagnostic Codes (ICD-9-CM)** |
| --- | --- | --- |
| **DEPRESSION** | **F33** |  |
| **Major Depressive Disorder**  Major depressive disorder, recurrent, mild  Major depressive disorder, recurrent, moderate  Major depressive disorder, recurrent severe without psychotic features  Major depressive disorder, recurrent, severe with psychotic symptoms  Major depressive disorder, recurrent, in remission  -Major depressive disorder, recurrent, in remission, unspecified  -Major depressive disorder, recurrent, in partial remission  -Major depressive disorder, recurrent, in full remission  Other recurrent depressive disorders  Major depressive disorder, recurrent, unspecified | F33.0  F33.1  F33.2  F33.3  F33.4  -F33.40  -F33.41  -F33.42  F33.8  F33.9 | 311.0 (depressive disorder, unspecified) |
| **ANXIETY** | **F41** |  |
| **Anxiety Disorder**  Panic disorder  Generalized anxiety disorder  Mixed anxiety and depressive disorder  Other mixed anxiety disorders  Other specified anxiety disorders  Anxiety disorder, unspecified | F41.0  F41.1  F41.2  F41.3  F41.8  F41.9 | 300.0 (anxiety states, unspecified) |
| **ALCOHOL** | **F10** |  |
| **Alcohol Use Disorder**  *Abuse*  Uncomplicated  With induced mood disorder  With induced psychotic disorder  - With delusions  - With hallucinations  - Unspecified  With other induced disorder  - Anxiety disorder  - Sexual disorder  - Sleep disorder  - Other induced disorder  With unspecified induced disorder  *Dependence*  Uncomplicated  With withdrawal  - Uncomplicated  - Delirium  - With perceptual disturbance  - Unspecified  With induced mood disorder  With induced psychotic disorder  - With delusions  - With hallucinations  - Unspecified  With induced persisting amnestic disorder  With induced persisting dementia  With other induced disorders  - Anxiety disorder  - Sexual dysfunction  - Sleep disorder  - Other induced disorder  With unspecified induced disorder  *Use, unspecified*  With withdrawal  - Uncomplicated  - Delirium  - With perceptual disturbance  - Unspecified  With induced mood disorder  With induced psychotic disorder  - Delusions  - Hallucinations  - Sleep disorder  With induced persisting amnestic disorder  With induced persisting dementia  With other induced disorders  - Anxiety disorder  - Sexual dysfunction  - Sleep disorder  - Other induced disorder  With unspecified induced disorder | *F10.1*  F10.10  F10.14  F10.15  - F10.150  - F10.151  - F10.159  F10.18  - F10.180  - F10.181  - F10.182  - F10.188  F10.19  *F10.2*  F10.20  F10.23  - F10.230  - F10.231  - F10.232  - F10.239  F10.24  F10.25  - F10.250  - F10.251  - F10.259  F10.26  F10.27  F10.28  - F10.280  - F10.281  - F10.282  - F10.288  F10.29  *F10.9*  F10.93  - F10.930  - F10.931  - F10.932  - F10.939  F10.94  F10.95  - F10.950  - F10.951  - F10.959  F10.96  F10.97  F10.98  - F10.980  - F10.981  - F10.982  - F10.983  F10.99 | 305.0 (Alcohol abuse, unspecified)  303.90 (unspecified alcohol dependence) |
| **Alcohol Intoxication**  *Abuse with intoxication (i.e. – intoxication with mild use disorder)*  - Uncomplicated  - Delirium  - With perceptual disturbance  - Unspecified  *Dependence with intoxication (i.e. – intoxication with mod- severe use disorder)*  - Uncomplicated  - Delirium  - With perceptual disturbance  - Unspecified  *Use with intoxication (i.e. – intoxication without use disorder)*  - Uncomplicated  - Delirium  - With perceptual disturbance  - Unspecified | *F10.12*  - F10.120  - F10.121  - F10.122  - F10.129  *F10.22*  - F10.220  - F10.221  - F10.222  - F10.229  *F10.92*  F10.920  F10.921  F10.922  F10.929 | 303.0 (alcohol dependence with unspecified acute alcohol intoxication) |
| **Other alcohol related medical disease**  Alcohol induced acute pancreatitis  - Without necrosis or infection  - With uninfected necrosis  - With infected necrosis  Alcohol induced chronic pancreatitis  Alcoholic myopathy  Degeneration of nervous system due to alcohol  Alcoholic polyneuropathy  Alcoholic cardiomyopathy  Alcoholic gastritis  - Without bleeding  - With bleeding  Alcoholic fatty liver  Alcoholic hepatitis  - Without ascites  - With ascites  Alcoholic fibrosis and sclerosis of liver  Alcoholic cirrhosis of liver  - Without ascites  - With ascites  Alcoholic hepatic failure  - Without coma  - With coma  Alcoholic liver disease, unspecified | K85.2  - K85.20  - K85.21  - K85.21  K86.0  G72.1  G31.2  G62.1  I42.6  K29.2  - K29.20  - K29.21  K70.0  K70.1  - K70.10  - K70.11  K70.2  K70.3  K70.30  K70.31  K70.4  - K70.40  - K70.41  K70.9 |  |
| **OPIOIDS** | **F11** |  |
| **Opioid Use Disorder**  *Abuse*  Uncomplicated  With induced mood disorder  With induced psychotic disorder  - With delusions  - With hallucinations  - Unspecified  With other induced disorder  - Anxiety disorder  - Sexual disorder  - Sleep disorder  - Other induced disorder  With unspecified induced disorder  *Dependence*  Uncomplicated  With withdrawal  - Uncomplicated  - Delirium  - With perceptual disturbance  - Unspecified  With induced mood disorder  With induced psychotic disorder  - With delusions  - With hallucinations  - Unspecified  With induced persisting amnestic disorder  With induced persisting dementia  With other induced disorders  - Anxiety disorder  - Sexual dysfunction  - Sleep disorder  - Other induced disorder  With unspecified induced disorder  *Use, unspecified*  With withdrawal  - Uncomplicated  - Delirium  - With perceptual disturbance  - Unspecified  With induced mood disorder  With induced psychotic disorder  - Delusions  - Hallucinations  - Sleep disorder  With induced persisting amnestic disorder  With induced persisting dementia  With other induced disorders  - Anxiety disorder  - Sexual dysfunction  - Sleep disorder  - Other induced disorder  With unspecified induced disorder | *F11.1*  F11.10  F11.14  F11.15  - F11.150  - F11.151  - F11.159  F11.18  - F11.180  - F11.181  - F11.182  - F11.188  F11.19  *F11.2*  F11.20  F11.23  - F11.230  - F11.231  - F11.232  - F11.239  F11.24  F11.25  - F11.250  - F11.251  - F11.259  F11.26  F11.27  F11.28  - F11.280  - F11.281  - F11.282  - F11.288  F11.29  *F11.9*  F11.93  - F11.930  - F11.931  - F11.932  - F11.939  F11.94  F11.95  - F11.950  - F11.951  - F11.959  F11.96  F11.97  F11.98  - F11.980  - F11.981  - F11.982  - F11.983  F11.99 | 305.5 (Opioid abuse, unspecified)  304.0 (Opioid dependence, unspecified)  292.0 (with withdrawal) |
| **Opioid Intoxication**  *Abuse with intoxication (i.e. – intoxication with mild use disorder)*  - Uncomplicated  - Delirium  - With perceptual disturbance  - Unspecified  *Dependence with intoxication (i.e. – intoxication with mod- severe use disorder)*  - Uncomplicated  - Delirium  - With perceptual disturbance  - Unspecified  *Use with intoxication (i.e. – intoxication without use disorder)*  - Uncomplicated  - Delirium  - With perceptual disturbance  - Unspecified | *F11.12*  - F11.120  - F11.121  - F11.122  - F11.129  *F11.22*  - F11.220  - F11.221  - F11.222  - F11.229  *F11.92*  F11.920  F11.921  F11.922  F11.929 | 292.89 (other specified drug induced mental disorders) |
| **SEDATIVE, HYPNOTICS, ANXIOLYTICS** | **F13** |  |
| **Sedative, Hypnotic, or Anxiolytic Use Disorder**  *Abuse*  Uncomplicated  With induced mood disorder  With induced psychotic disorder  - With delusions  - With hallucinations  - Unspecified  With other induced disorder  - Anxiety disorder  - Sexual disorder  - Sleep disorder  - Other induced disorder  With unspecified induced disorder  *Dependence*  Uncomplicated  With withdrawal  - Uncomplicated  - Delirium  - With perceptual disturbance  - Unspecified  With induced mood disorder  With induced psychotic disorder  - With delusions  - With hallucinations  - Unspecified  With induced persisting amnestic disorder  With induced persisting dementia  With other induced disorders  - Anxiety disorder  - Sexual dysfunction  - Sleep disorder  - Other induced disorder  With unspecified induced disorder  *Use, unspecified*  With withdrawal  - Uncomplicated  - Delirium  - With perceptual disturbance  - Unspecified  With induced mood disorder  With induced psychotic disorder  - Delusions  - Hallucinations  - Sleep disorder  With induced persisting amnestic disorder  With induced persisting dementia  With other induced disorders  - Anxiety disorder  - Sexual dysfunction  - Sleep disorder  - Other induced disorder  With unspecified induced disorder | *F13.1*  F13.10  F13.14  F13.15  - F13.150  - F13.151  - F13.159  F13.18  - F13.180  - F13.181  - F13.182  - F13.188  F13.19  *F13.2*  F13.20  F13.23  - F13.230  - F13.231  - F13.232  - F13.239  F13.24  F13.25  - F13.250  - F13.251  - F13.259  F13.26  F13.27  F13.28  - F13.280  - F13.281  - F13.282  - F13.288  F13.29  *F13.9*  F13.93  - F13.930  - F13.931  - F13.932  - F13.939  F13.94  F13.95  - F13.950  - F13.951  - F13.959  F13.96  F13.97  F13.98  - F13.980  - F13.981  - F13.982  - F13.983  F13.99 | 305.4 (sedative, hypnotic, or anxiolytic dependence, unspecified)  304.1 (sedative, hypnotic, or anxiolytic dependence, unspecified) |
| **Sedative, Hypnotic, or Anxiolytic Intoxication**  *Abuse with intoxication (i.e. – intoxication with mild use disorder)*  - Uncomplicated  - Delirium  - With perceptual disturbance  - Unspecified  *Dependence with intoxication (i.e. – intoxication with mod- severe use disorder)*  - Uncomplicated  - Delirium  - With perceptual disturbance  - Unspecified  *Use with intoxication (i.e. – intoxication without use disorder)*  - Uncomplicated  - Delirium  - With perceptual disturbance  - Unspecified | *F13.12*  - F13.120  - F13.121  - F13.122  - F13.129  *F13.22*  - F13.220  - F13.221  - F13.222  - F13.229  *F13.92*  F13.920  F13.921  F13.922  F13.929 |  |
| **COCAINE** | **F14** |  |
| **Cocaine Use Disorder**  *Abuse*  Uncomplicated  With induced mood disorder  With induced psychotic disorder  - With delusions  - With hallucinations  - Unspecified  With other induced disorder  - Anxiety disorder  - Sexual disorder  - Sleep disorder  - Other induced disorder  With unspecified induced disorder  *Dependence*  Uncomplicated  With withdrawal  - Uncomplicated  - Delirium  - With perceptual disturbance  - Unspecified  With induced mood disorder  With induced psychotic disorder  - With delusions  - With hallucinations  - Unspecified  With induced persisting amnestic disorder  With induced persisting dementia  With other induced disorders  - Anxiety disorder  - Sexual dysfunction  - Sleep disorder  - Other induced disorder  With unspecified induced disorder  *Use, unspecified*  With withdrawal  - Uncomplicated  - Delirium  - With perceptual disturbance  - Unspecified  With induced mood disorder  With induced psychotic disorder  - Delusions  - Hallucinations  - Sleep disorder  With induced persisting amnestic disorder  With induced persisting dementia  With other induced disorders  - Anxiety disorder  - Sexual dysfunction  - Sleep disorder  - Other induced disorder  With unspecified induced disorder | *F14.1*  F14.10  F14.14  F14.15  - F14.150  - F14.151  - F14.159  F14.18  - F14.180  - F14.181  - F14.182  - F14.188  F14.19  *F14.2*  F14.20  F14.23  - F14.230  - F14.231  - F14.232  - F14.239  F14.24  F14.25  - F14.250  - F14.251  - F14.259  F14.26  F14.27  F14.28  - F14.280  - F14.281  - F14.282  - F14.288  F14.29  *F14.9*  F14.93  - F14.930  - F14.931  - F14.932  - F14.939  F14.94  F14.95  - F14.950  - F14.951  - F14.959  F14.96  F14.97  F14.98  - F14.980  - F14.981  - F14.982  - F14.983  F14.99 | 305.6 (cocaine abuse, unspecified)  304.2 (cocaine dependence, unspecified) |
| **Cocaine Intoxication**  *Abuse with intoxication (i.e. – intoxication with mild use disorder)*  - Uncomplicated  - Delirium  - With perceptual disturbance  - Unspecified  *Dependence with intoxication (i.e. – intoxication with mod- severe use disorder)*  - Uncomplicated  - Delirium  - With perceptual disturbance  - Unspecified  *Use with intoxication (i.e. – intoxication without use disorder)*  - Uncomplicated  - Delirium  - With perceptual disturbance  - Unspecified | *F14.12*  - F14.120  - F14.121  - F14.122  - F14.129  *F14.22*  - F14.220  - F14.221  - F14.222  - F14.229  *F14.92*  F14.920  F14.921  F14.922  F14.929 |  |
| **AMPHETAMINES** | **F15 (Stimulants)** |  |
| **Amphetamine Use Disorder**  *Abuse*  Uncomplicated  With induced mood disorder  With induced psychotic disorder  - With delusions  - With hallucinations  - Unspecified  With other induced disorder  - Anxiety disorder  - Sexual disorder  - Sleep disorder  - Other induced disorder  With unspecified induced disorder  *Dependence*  Uncomplicated  With withdrawal  - Uncomplicated  - Delirium  - With perceptual disturbance  - Unspecified  With induced mood disorder  With induced psychotic disorder  - With delusions  - With hallucinations  - Unspecified  With induced persisting amnestic disorder  With induced persisting dementia  With other induced disorders  - Anxiety disorder  - Sexual dysfunction  - Sleep disorder  - Other induced disorder  With unspecified induced disorder  *Use, unspecified*  With withdrawal  - Uncomplicated  - Delirium  - With perceptual disturbance  - Unspecified  With induced mood disorder  With induced psychotic disorder  - Delusions  - Hallucinations  - Sleep disorder  With induced persisting amnestic disorder  With induced persisting dementia  With other induced disorders  - Anxiety disorder  - Sexual dysfunction  - Sleep disorder  - Other induced disorder  With unspecified induced disorder | *F15.1*  F15.10  F15.14  F15.15  - F15.150  - F15.151  - F15.159  F15.18  - F15.180  - F15.181  - F15.182  - F15.188  F15.19  *F15.2*  F15.20  F15.23  - F15.230  - F15.231  - F15.232  - F15.239  F15.24  F15.25  - F15.250  - F15.251  - F15.259  F15.26  F15.27  F15.28  - F15.280  - F15.281  - F15.282  - F15.288  F15.29  *F15.9*  F15.93  - F15.930  - F15.931  - F15.932  - F15.939  F15.94  F15.95  - F15.950  - F15.951  - F15.959  F15.96  F15.97  F15.98  - F15.980  - F15.981  - F15.982  - F15.983  F15.99 | 305.7 (amphetamine abuse, unspecified)  304.4 (amphetamine dependence, unspecified) |
| **Amphetamine Intoxication**  *Abuse with intoxication (i.e. – intoxication with mild use disorder)*  - Uncomplicated  - Delirium  - With perceptual disturbance  - Unspecified  *Dependence with intoxication (i.e. – intoxication with mod- severe use disorder)*  - Uncomplicated  - Delirium  - With perceptual disturbance  - Unspecified  *Use with intoxication (i.e. – intoxication without use disorder)*  - Uncomplicated  - Delirium  - With perceptual disturbance  - Unspecified | *F15.12*  - F15.120  - F15.121  - F15.122  - F15.129  *F15.22*  - F15.220  - F15.221  - F15.222  - F15.229  *F15.92*  F15.920  F15.921  F15.922  F15.929 |  |
| **OTHER HALLUCINOGENS & PHENCYCLIDINE** | **F16** |  |
| **Other Hallucinogen & Phencyclidine Use Disorder**  *Abuse*  Uncomplicated  With induced mood disorder  With induced psychotic disorder  - With delusions  - With hallucinations  - Unspecified  With other induced disorder  - Anxiety disorder  - Sexual disorder  - Sleep disorder  - Other induced disorder  With unspecified induced disorder  *Dependence*  Uncomplicated  With withdrawal  - Uncomplicated  - Delirium  - With perceptual disturbance  - Unspecified  With induced mood disorder  With induced psychotic disorder  - With delusions  - With hallucinations  - Unspecified  With induced persisting amnestic disorder  With induced persisting dementia  With other induced disorders  - Anxiety disorder  - Sexual dysfunction  - Sleep disorder  - Other induced disorder  With unspecified induced disorder  *Use, unspecified*  With withdrawal  - Uncomplicated  - Delirium  - With perceptual disturbance  - Unspecified  With induced mood disorder  With induced psychotic disorder  - Delusions  - Hallucinations  - Sleep disorder  With induced persisting amnestic disorder  With induced persisting dementia  With other induced disorders  - Anxiety disorder  - Sexual dysfunction  - Sleep disorder  - Other induced disorder  With unspecified induced disorder | *F16.1*  F16.10  F16.14  F16.15  - F16.150  - F16.151  - F16.159  F16.18  - F16.180  - F16.181  - F16.182  - F16.188  F16.19  *F16.2*  F16.20  F16.23  - F16.230  - F16.231  - F16.232  - F16.239  F16.24  F16.25  - F16.250  - F16.251  - F16.259  F16.26  F16.27  F16.28  - F16.280  - F16.281  - F16.282  - F16.288  F16.29  *F16.9*  F16.93  - F16.930  - F16.931  - F16.932  - F16.939  F16.94  F16.95  - F16.950  - F16.951  - F16.959  F16.96  F16.97  F16.98  - F16.980  - F16.981  - F16.982  - F16.983  F16.99 | 305.3 (hallucinogen abuse, unspecified)  304.5 (hallucinogen dependence, unspecified) |
| **Other Hallucinogen & Phencyclidine Intoxication**  *Abuse with intoxication (i.e. – intoxication with mild use disorder)*  - Uncomplicated  - Delirium  - With perceptual disturbance  - Unspecified  *Dependence with intoxication (i.e. – intoxication with mod- severe use disorder)*  - Uncomplicated  - Delirium  - With perceptual disturbance  - Unspecified  *Use with intoxication (i.e. – intoxication without use disorder)*  - Uncomplicated  - Delirium  - With perceptual disturbance  - Unspecified | *F16.12*  - F16.120  - F16.121  - F16.122  - F16.129  *F16.22*  - F16.220  - F16.221  - F16.222  - F16.229  *F16.92*  F16.920  F16.921  F16.922  F16.929 |  |
| **INHALANT** | **F18** |  |
| **Inhalant Use Disorder**  *Abuse*  Uncomplicated  With induced mood disorder  With induced psychotic disorder  - With delusions  - With hallucinations  - Unspecified  With other induced disorder  - Anxiety disorder  - Sexual disorder  - Sleep disorder  - Other induced disorder  With unspecified induced disorder  *Dependence*  Uncomplicated  With withdrawal  - Uncomplicated  - Delirium  - With perceptual disturbance  - Unspecified  With induced mood disorder  With induced psychotic disorder  - With delusions  - With hallucinations  - Unspecified  With induced persisting amnestic disorder  With induced persisting dementia  With other induced disorders  - Anxiety disorder  - Sexual dysfunction  - Sleep disorder  - Other induced disorder  With unspecified induced disorder  *Use, unspecified*  With withdrawal  - Uncomplicated  - Delirium  - With perceptual disturbance  - Unspecified  With induced mood disorder  With induced psychotic disorder  - Delusions  - Hallucinations  - Sleep disorder  With induced persisting amnestic disorder  With induced persisting dementia  With other induced disorders  - Anxiety disorder  - Sexual dysfunction  - Sleep disorder  - Other induced disorder  With unspecified induced disorder | *F18.1*  F18.10  F18.14  F18.15  - F18.150  - F18.151  - F18.159  F18.18  - F18.180  - F18.181  - F18.182  - F18.188  F18.19  *F18.2*  F18.20  F18.23  - F18.230  - F18.231  - F18.232  - F18.239  F18.24  F18.25  - F18.250  - F18.251  - F18.259  F18.26  F18.27  F18.28  - F18.280  - F18.281  - F18.282  - F18.288  F18.29  *F18.9*  F18.93  - F18.930  - F18.931  - F18.932  - F18.939  F18.94  F18.95  - F18.950  - F18.951  - F18.959  F18.96  F18.97  F18.98  - F18.980  - F18.981  - F18.982  - F18.983  F18.99 | 305.9 (other drug abuse, unspecified)  304.6 (other specified drug dependence, unspecified) |
| **Inhalant Intoxication**  *Abuse with intoxication (i.e. – intoxication with mild use disorder)*  - Uncomplicated  - Delirium  - With perceptual disturbance  - Unspecified  *Dependence with intoxication (i.e. – intoxication with mod- severe use disorder)*  - Uncomplicated  - Delirium  - With perceptual disturbance  - Unspecified  *Use with intoxication (i.e. – intoxication without use disorder)*  - Uncomplicated  - Delirium  - With perceptual disturbance  - Unspecified | *F18.12*  - F18.120  - F18.121  - F18.122  - F18.129  *F18.22*  - F18.220  - F18.221  - F18.222  - F18.229  *F18.92*  F18.920  F18.921  F18.922  F18.929 |  |
| **POLYSUBSTANCE & OTHER OR UNKNOWN** | **F19 (multiple drug)** |  |
| **Polysubstance & Other or Unknown Use Disorder**  *Abuse*  Uncomplicated  With induced mood disorder  With induced psychotic disorder  - With delusions  - With hallucinations  - Unspecified  With other induced disorder  - Anxiety disorder  - Sexual disorder  - Sleep disorder  - Other induced disorder  With unspecified induced disorder  *Dependence*  Uncomplicated  With withdrawal  - Uncomplicated  - Delirium  - With perceptual disturbance  - Unspecified  With induced mood disorder  With induced psychotic disorder  - With delusions  - With hallucinations  - Unspecified  With induced persisting amnestic disorder  With induced persisting dementia  With other induced disorders  - Anxiety disorder  - Sexual dysfunction  - Sleep disorder  - Other induced disorder  With unspecified induced disorder  *Use, unspecified*  With withdrawal  - Uncomplicated  - Delirium  - With perceptual disturbance  - Unspecified  With induced mood disorder  With induced psychotic disorder  - Delusions  - Hallucinations  - Sleep disorder  With induced persisting amnestic disorder  With induced persisting dementia  With other induced disorders  - Anxiety disorder  - Sexual dysfunction  - Sleep disorder  - Other induced disorder  With unspecified induced disorder | *F19.1*  F19.10  F19.14  F19.15  - F19.150  - F19.151  - F19.159  F19.18  - F19.180  - F19.181  - F19.182  - F19.188  F19.19  *F19.2*  F19.20  F19.23  - F19.230  - F19.231  - F19.232  - F19.239  F19.24  F19.25  - F19.250  - F19.251  - F19.259  F19.26  F19.27  F19.28  - F19.280  - F19.281  - F19.282  - F19.288  F19.29  *F19.9*  F19.93  - F19.930  - F19.931  - F19.932  - F19.939  F19.94  F19.95  - F19.950  - F19.951  - F19.959  F19.96  F19.97  F19.98  - F19.980  - F19.981  - F19.982  - F19.983  F19.99 | 305.9 (mixed or other drug abuse, unspecified)  304.7, 304.8, 304.9 (combination dependence unspecified with and without opioids, unspecified drug dependence) |
| **Polysubstance & Other or Unknown Intoxication**  *Abuse with intoxication (i.e. – intoxication with mild use disorder)*  - Uncomplicated  - Delirium  - With perceptual disturbance  - Unspecified  *Dependence with intoxication (i.e. – intoxication with mod- severe use disorder)*  - Uncomplicated  - Delirium  - With perceptual disturbance  - Unspecified  *Use with intoxication (i.e. – intoxication without use disorder)*  - Uncomplicated  - Delirium  - With perceptual disturbance  - Unspecified | *F19.12*  - F19.120  - F19.121  - F19.122  - F19.129  *F19.22*  - F19.220  - F19.221  - F19.222  - F19.229  *F19.92*  F19.920  F19.921  F19.922  F19.929 |  |
